# Editorial: Human-centered AI: Crowd computing

**DOI:** 10.3389/frai.2023.1161006

**Published:** 2023-03-15

**Authors:** Jie Yang, Alessandro Bozzon, Ujwal Gadiraju, Matthew Lease

**Affiliations:** ^1^Web Information Systems, Delft University of Technology, Delft, Netherlands; ^2^Knowledge and Intelligence Design, Delft University of Technology, Delft, Netherlands; ^3^School of Information, The University of Texas at Austin, Austin, TX, United States

**Keywords:** human-centered AI, human-in-the-loop AI, human-AI interaction, human computation, crowdsourcing

## 1. Introduction

Human computation (HCOMP) and crowdsourcing (Law and von Ahn, 2011; Quinn and Bederson, 2011; Kittur et al., 2013; Lease and Alonso, 2018) have been instrumental to advances seen in artificial intelligence (AI) and machine learning (ML) over the past 15+ years. AI/ML has an insatiable hunger for human labeled training to supervise models, with training data scale playing a significant (if not dominant) role in driving the predictive performance of models (Halevy et al., 2009). The centrality of such human-labeled data to the success and continuing advancement of AI/ML is thus at the heart of today's *data-centric AI* movement (Mazumder et al., 2022). Moreover, recent calls for *data excellence* (Aroyo et al., 2022) reflect growing recognition that AI/ML data scale alone does not suffice. The quality of human labeled data also plays a tremendous role in AI/ML success, and ignoring this can be perilous to deployed AI/ML systems (Sambasivan et al., 2021), as prominent, public failures have shown.

HOMP and crowdsourcing have also enabled hybrid, human-in-the-loop, crowd-powered computing (Demartini et al., 2017). When state-of-the-art AI/ML cannot provide sufficient capabilities or predictive performance to meet practical needs for real-world deployment, hybrid systems utilize HCOMP at run-time to deliver last-mile capabilities where AI/ML fall short (Gadiraju and Yang, 2020). This has enabled a new class of innovative and more capable applications, systems, and companies to be built (Barr and Cabrera, 2006). While work in HCOMP is centuries old (Grier, 2013), access to an increasingly Internet-connected and well-educated world population led to the advent of crowdsourcing (Howe, 2006). This has allowed AI/ML systems to call on human help at run-time as “Human Processing Units (HPUs)”(Davis et al., 2010), “Remote Person Calls (RPCs)” (Bederson and Quinn, 2011), and “the Human API” (Irani and Silberman, 2013).

Across both data labeling and run-time HCOMP, crowdsourcing has enabled AI/ML builders to tap into the “wisdom of the crowd” (Surowiecki, 2005) and harness *collective intelligence* from large groups of people. As AI/ML systems have grown both more powerful and ubiquitous, appreciation of their capabilities has also been tempered by concerns of prevalence and propagation of biases, lack of robustness, fairness, and transparency as well as ethical and societal implications. At the same time, crowdsourced access to a global, diverse set of contributors provides an incredible avenue to boost inclusivity and fairness in both AI/ML labeled datasets and hybrid, human-in-the-loop systems. However, important questions remain about the roles and treatment of AI/ML data workers, and the extent to which AI/ML advances are creating new economic opportunities for human workers (Paritosh et al., 2011) or exploiting hidden human labor (Bederson and Quinn, 2011; Fort et al., 2011; Irani and Silberman, 2013; Lease and Alonso, 2018; Gray and Suri, 2019). This has prompted the development of ethical principles for crowd work (Graham et al., 2020) and calls for *responsible sourcing* of AI/ML data (Partnership on AI, 2021).

As the above discussion reflects, HCOMP and crowdsourcing reflects a rich amalgamation of interdisciplinary research. In particular, the confluence of two key research communities—AI/ML and human-computer interaction (HCI)—has been central to founding and advancing HCOMP and crowdsourcing. Beyond this, related work draws upon a wide and rich body of diverse areas, including (but not limited to) computational social science, digital humanities, economics, ethics, law / policy / regulation, and social computing. More broadly, the HCOMP and crowdsourcing community promotes the exchange of advances in the state-of-the-art and best practices not only among researchers but also engineers and practitioners, to encourage dialogue across disciplines and communities of practice.

## 2. Call for papers: Aim and scope

Our organization of this Frontiers *Research Topic* called for new and high-impact contributions in HCOMP and crowdsourcing. We especially encouraged work that generates new insights into the collaboration and interaction between humans and AI, enlarging understanding about hybrid human-in-the-loop and algorithm-in-the-loop systems (Green and Chen, 2020). This includes human-AI interaction, algorithmic and interface techniques for augmenting human abilities to AI systems. It also spans issues that affect how humans collaborate and interact with AI systems such as bias, interpretability, usability, and trustworthiness. We welcomed both system-centered and human-centered approaches to human+AI systems, considering humans as users and stakeholders, or as active contributors and an integral part of the system.

Our call for papers invited submissions relevant to theory, studies, tools and/or applications that present novel, interesting, impactful interactions between people and computational systems. These cover a broad range of scenarios across human computation, wisdom of the crowds, crowdsourcing, and people-centric AI methods, systems and applications.

The scope of the Research Topics included the following themes:

- Crowdsourcing applications and techniques.
- Techniques that enable and enhance human-in-the-loop systems, making them more efficient, accurate, and human-friendly.
- Studies about how people perform tasks individually, in groups, or as a crowd.
- Approaches to make crowd science FAIR (Findable, Accessible, Interoperable, Reproducible) and studies assessing and commenting on the FAIRness of human computation and crowdsourcing practice.
- Studies into fairness, accountability, transparency, ethics, and policy implications for crowdsourcing and human computation.
- Methods that use human computation and crowdsourcing to build people-centric AI systems and applications, including topics such as reliability, interpretability, usability, and trustworthiness.
- Studies into the reliability and other quality aspects of human-annotated and -curated datasets, especially for AI systems.
- Studies about how people and intelligent systems interact and collaborate with each other and studies revealing the influences and impact of intelligent systems on society.
- Crowdsourcing studies into the socio-technical aspects of AI systems: privacy, bias, and trust.

## 3. Partnership with AAAI HCOMP

For over a decade, the premier venue for disseminating the latest research findings on HCOMP and crowdsourcing has been the Association for the Advancement of Artificial Intelligence (AAAI) Conference on Human Computation and Crowdsourcing (AAAI HCOMP).^1^ Early HCOMP workshops at KDD and AAAI conferences (2009-2012) led to the genesis of the AAAI HCOMP conference in 2013. To further strengthen this Frontiers *Research Topic*, we partnered with AAAI HCOMP to invite submissions; papers accepted to HCOMP 2021 were offered a streamlined process for publication in this topic (e.g., maintaining the same reviewers when possible). We accepted four such submissions that extend earlier HCOMP 2021 papers (Samiotis et al., 2021; Welty et al., 2021; Yamanaka, 2021; Yasmin et al., 2021).

## 4. Managing conflicts-of-interest (COI)

“The Frontiers review system is designed to guarantee the most transparent and objective editorial and review process, and because the handling editor's and reviewers' names are made public upon the publication of articles, conflicts of interest will be widely apparent” (Frontiers, 2023). For this Frontiers *Research Topic*, two submissions from topic editors were routed by Frontiers staff to other editors not otherwise associated with this *Research Topic* and had no COI with the topic editors. Both submissions were ultimately accepted (Pradhan et al.; Samiotis et al.), after which the identity of each handling editor became publicly available. We thank these additional editors for their contributions to this *Research Topic*.

## 5. Research Topic contributions

A total of nine articles were accepted, contributing studies into factors of human computation and crowdsourcing, to their applications to human-AI collaborative systems and large-scale behavioral studies. In the following, we very briefly summarize these works.

### 5.1. Quality in crowdsourced data annotation

Annotation quality is often a key concern in crowdsourced labeling. Pradhan et al. introduce a three-stage FIND-RESOLVE-LABEL workflow to reduce ambiguity in annotation task instructions. Their workflow allows workers to provide feedback on ambiguous task instructions to a requester. Another aspect of annotation quality is worker disagreement, for which a number of methods have been developed. Drawing from the observation that the effectiveness of annotation depends on the level of noise in the data, Uma et al. investigate the use of temperature scaling to estimate noise. Yasmin et al. investigates the effect of different forms of input elicitation to improve the quality of inferred labels in image classification, suggesting that more accurate results can be achieved when labels and self-reported confidence are used as features for classifiers.

### 5.2. Human-centered computation and interaction in AI

Tocchetti et al. study the effect of gamified activities to improve crowds' understanding of black-box models, addressing the intelligibility issue of explainable AI. They consider gamified activities to educate crowds by AI researchers. Yamanaka investigates the effectiveness of crowdsourcing for validating user performance models, especially the error-rate prediction model in target pointing tasks, which requires data from many repetitive experiments by participants for each task condition to measure the central tendency of the error rate. Welty et al. studies crowd knowledge creation for curating class-level knowledge graphs. Their three-tier crowd approach to elicit class-level attributes addresses the label sparsity problem faced by AI/ML systems.

### 5.3. Human factors in human computation

Vinella, Hu et al. focuses the effect of human agency in team formation on team performance. They found that in open collaboration scenarios, e.g., hackathon, teams formed by workers themselves are more competitive, compared to those formed by algorithms. Samiotis et al. explore the possession of musical skills in the worker population. Their study shows that untrained workers possess high perception skills that can be useful in many music annotation tasks. Vinella, Odo et al. study the effect of personality on task performance by ad-hoc teams composed of strangers, especially in solving critical tasks that are often time-bounded and high-stress, e.g., incident response. Their results identify personality traits that affect team performance and in addition to that, relevant communication patterns used by winning teams.

## Author contributions

All authors listed have made a substantial, direct, and intellectual contribution to the work and approved it for publication.

## Footnotes

^1^ https://humancomputation.com/
